# Do we need a 6D’s Framework of Nutritional Stewardship in critical care?

**DOI:** 10.1186/s44158-021-00009-4

**Published:** 2021-10-02

**Authors:** Dafne Pisani, Paolo Navalesi, Silvia De Rosa

**Affiliations:** 1 Department of Emergency and Organ Transplantation, University of Bari “Aldo Moro”, Bari, Italy; 2 Department of Medicine, Anesthesia and Critical Care Unit, Padua University Hospital, Padua, Italy; 3 Department of Anesthesiology and Intensive Care, San Bortolo Hospital, Vicenza, Italy

**Keywords:** Critical care, Nutritional status, Nutrition support, Nutrition stewardship

## Abstract

Recent European Society for Clinical Nutrition and Metabolism (ESPEN) guideline on clinical nutrition in the intensive care unit had as ultimate goal the achievement of optimal nutritional support for critically ill patients and to illuminate the gaps in knowledge in order to provide priorities for future clinical research. Although malnutrition is a vital part of the treatment of patients with critical illness and injury, nutrition in the critically ill is not one size fits all. Both clinical nutrition guidelines and ICU experts have recognized the need for a new, individualized approach to nutrition. Nutrition stewardship, analog to antimicrobial and fluid stewardship, could be defined as the “ongoing effort by a healthcare institution to optimise artificial nutrition use in order to improve patient outcomes, ensure cost effective therapy and reduce adverse sequelae.” A robust nutrition stewardship program could gain reputation if the concept will spread to various national programs and regulatory guidelines released in the recent past.

## Introduction

Recent European Society for Clinical Nutrition and Metabolism (ESPEN) guideline on clinical nutrition in the intensive care unit (ICU) had as ultimate goal the achievement of optimal nutritional support for ICU patients and to illuminate the gaps in knowledge in order to provide priorities for future clinical research [1]. The main goal is to attenuate the development of malnutrition. Although malnutrition is a vital part of the treatment of patients with critical illness and injury, nutrition in the critically ill is not one size fits all. Both clinical nutrition guidelines and ICU experts have recognized the need for a new, individualized approach to nutritional care. The input of experts, such as nutritionists, who are knowledgeable of nutritional assessment of the critically ill patient, the route of nutritional support, nutritional access, fluid and electrolyte issues, specialty enteral products, and optimal blood glucose control. Choosing the right enteral feeding formula may positively affect a patient's outcome; targeted use of therapeutic formulas can reduce the incidence of infectious complications, shorten lengths of stay in the ICU and in the hospital, and lower risk for mortality [2]. Optimizing the use of currently available enteral and parenteral nutrition is principally driven by their high cost and attendant complications. Nutrition stewardship, analog to antimicrobial [3] and fluid stewardshi p[4], could be defined as the “ongoing effort by a healthcare institution to optimise artificial nutrition use in order to improve patient outcomes, ensure cost effective therapy and reduce adverse sequelae”.

A robust nutrition stewardship program (NSP) could gain reputation if the concept will spread to various national programs and regulatory guidelines released in the recent past.

## Essential elements of nutrition stewardship program

### Implementation of nutrition guidelines

Practice guidelines are the starting point on the NSP roadmap. There are currently four international clinical practice guidelines available to inform the nutrition management of critically ill patients [1, 5–7].

The latest drafted are the ESPEN Guidelines (2019) [1] as an update of 2006 and 2009 editions. In ESPEN, the first 48 h are fundamental for inducing malnutrition in critically ill patients.

At present, malnutrition is considered a key element in reducing mortality, the duration of mechanical ventilation, and hospitalization times [8]. In particular, those patients who have to stay in the ICU for more than 7 days. A current meta-analysis with 20 studies involving 1168 patients, the percentage of malnutrition ranges from 38 to 78% [8]. There are no tools to estimate the nutritional status of the critically ill patient but tools to assess the nutritional risk (NUTRIC, mNUTRIC, SGA, MUST) [9]. It is important to initiate enteral feeding (EN) early via nasogastric tube or via percutaneous endoscopic gastrostomy or jejunostomy if after 4 weeks [10].

### Guidelines adapted to the local context

Guidelines for the optimal nutritional support are designed to assist clinicians in providing appropriate evidence-based care. However, a gap exists between research recommendations and actual practice despite the growing interest in implementation of nutritional therapy guidelines in critical care.

To assess the level of bedside adherence to clinical practice guidelines for enteral nutrition in critically ill patients receiving mechanical ventilation in 2010 was published a multicenter, prospective, observational study to evaluate professional practices and quantify the differences between what is recommended in clinical guidelines and what actually happens at the bedside. In this study, the relationship between clinician-delivered and guideline-prescribed calories was greater than 80% over the first week of hospitalization.

Most nutrition studies have investigated the acute phase of the disease in critically ill patients, but no benefits have been identified if EN is administered early. Three moments have been identified in ESPEN: early acute phase, late acute phase, and recovery phase within the first week, but it had already been shown that delaying the onset of PN results in an increase in the percentage of discharged and alive patients [10, 11].

However, it is clear that adaptation to local circumstances with input from senior clinicians is likely to increase acceptance rates.

### Prescription approval with post-prescription review and feedback

A pragmatic approach has been proposed by the new guidelines. Nutrition should be considered a support to basic therapies and not further pharmacotherapy, in order mainly to avoid iatrogenic damage, with the consent of nutritionists and not only of intensive care doctors. Restrictive intervention based on energy and protein goals in the early stages of a critical illness is more than three times more influential than persuasive interventions, such as education, on prescribing behavior. They may wonder if the current paradigm shift is just the pendulum of clinical guidelines and expert opinion swinging from left to right and vice versa, from early–generous to late–reluctant nutritional support.

### 6D’s Framework of Nutritional Stewardship

Personalized nutrition therapy, while respecting different targets during the phases of the patient journey after critical illness, should be prescribed and monitored.

A good nutrition management should be based, as in fluid management, on a series of coordinated interventions, introduced to select the optimal type of nutrition, dose, and duration of therapy in order to obtain a better clinical outcome, prevention of adverse events, and reduction costs. This can be summarized by the 6D letters—diagnosis, drug, dose, duration, de-escalation, and discharg e[12].

#### D for diagnosis

Identifying patients at risk of malnutrition is the first step in the nutritional care process within a multimodal care system. Early identification of patients at risk of malnutrition or who are malnourished is crucial in order to start a timely and adequate nutritional support. In this initial phase, the goal should be to provide a nutritional support and minimize the loss of lean body mass. For this reason, nutritional screening and assessment should take place in every patient in the ICU. Nutritional risk screening with simple and rapid tools should be performed systematically in each patient at ICU admission [13, 14]. Comprehensive detailed nutritional assessment for a nutritional care plan should be performed thereafter in those patients identified as at risk of malnutrition or who are malnourished [14]. This screening should be performed using an interdisciplinary approach in collaboration with a dietician using subjective and objective parameters such as clinical history, physical examination, body composition measurements, functional assessment, and laboratory values. Systematic nutritional risk screening and standardized nutritional management may also contribute to reduced healthcare costs.

#### D for Drug

Nutrients are the “chemical intelligence” that your body needs to be healthy. All chemical reactions in the body are catalyzed by enzymes. A nutrient, also called “co-factors” or “co-enzymes,” is a metabolic enzyme activator that we needed to make our body work properly. For example, Arginine is an amino acid derived from proteins which activates an enzyme called Nitric Oxide Synthase that can dilate blood vessels and decrease blood pressure.

**Fig. 1:**
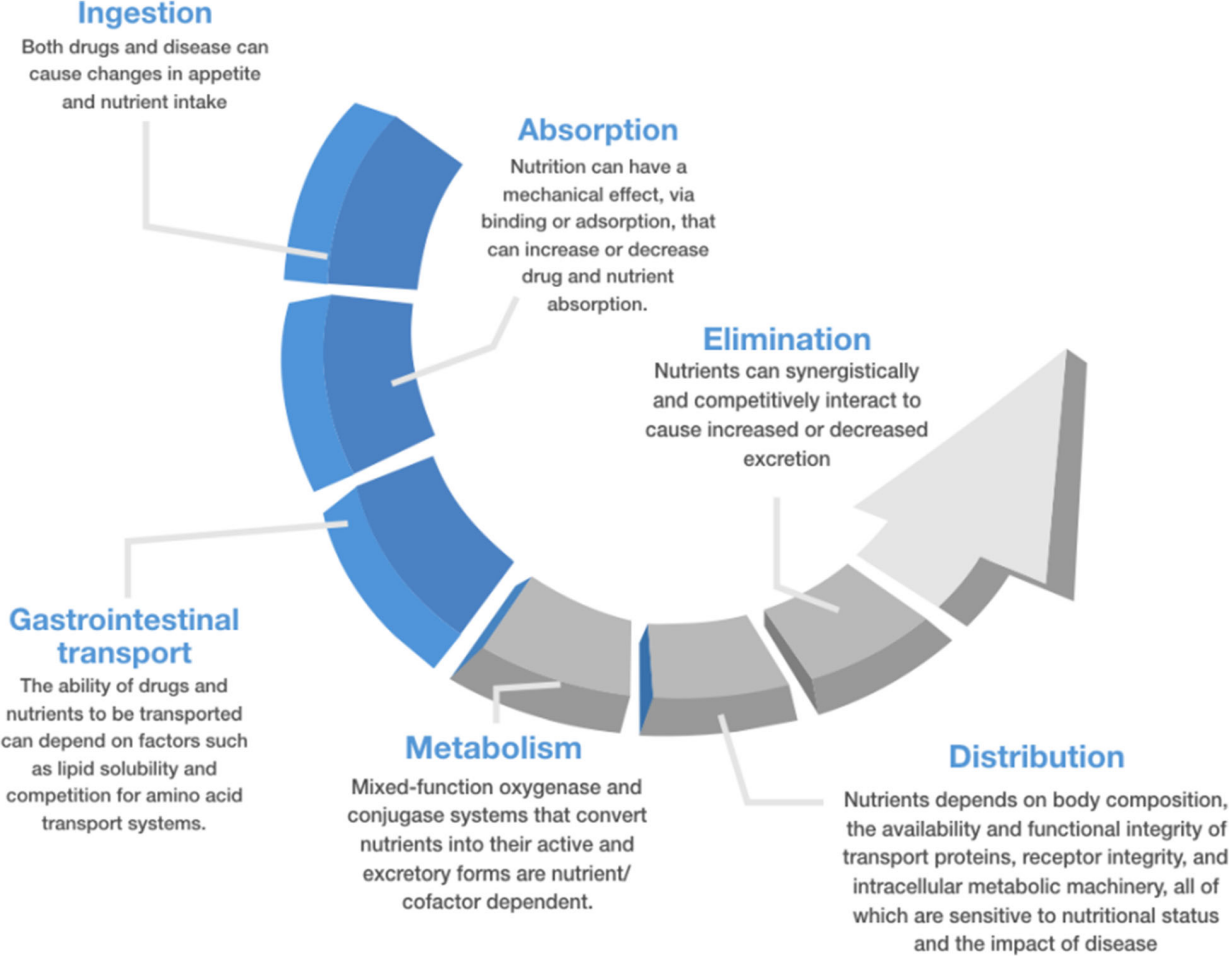
Potential mechanism of nutrients-drugs interaction

Nutrition may be defined as the sum of the processes involved in the taking in and use of food substances through which growth, repair, and maintenance of activities of the body as a whole or in any of its parts are accomplished. The processes of nutrition consist of ingestion, digestion, absorption, metabolism, functional use/activation of dependent systems, and excretion. All these processes are similarly integral to how the body takes in and uses therapeutics/drugs. Nutrients, similarly to drugs, act upon metabolic enzymes. Not only do drugs and nutrients share these same processes, their availability and function are also intimately and inextricably entwined [15] (Fig. 1).

Each kind of nutrition presents a different compound but also a different quantity of calories, nitrogen, protein, glucose, lipids, and micronutrients. For example, enteral feeding formulas fall into several general categories, such as polymeric formulas, feeding modules, elemental, and specialized or disease-specific formulas. For this reason, for each type of nutrition, there is a specific indication, contraindication, and potential adverse effect [16].

#### D for dose

All patients should be guaranteed the amount of nutrition that prevents accelerated depletion. The nutritional requirements of the critically ill are made up of the following important components: total energy, protein, lipids, carbohydrates, and micronutrients.

“Only the dose permits something not to be poisonous”. Like other drugs, it is the dose of nutrition that makes them poisonous. Thus, choosing the right dose implies that we take into account the pharmacokinetics and pharmacodynamics, as well as volume kinetics, since nutrition may also contribute to fluid accumulation [13]. Within the context of pharmacokinetics and pharmacodynamics, several pathways exist by which nutrition might affect drugs and vice versa (Fig. 1) and it should be considered.

#### D for duration

The duration of total or supplemental artificial nutritional therapy is important in critical illness, particularly, in case of transition from PN to EN, based on a specific nutritional care plan. Like for antibiotics, the duration of artificial therapy, particularly in the case of parenteral nutrition, must be as short as possible to allow. However, many clinicians use certain triggers to start, but are unaware of triggers to stop artificial nutrition, increasing the potential of complications if a nutritional plan is not well performed. Every unphysiologic intervention of artificial nutrition in critically ill patients may evoke complications and side effects and can prolong artificial nutrition and ICU stay [17].

#### D for de-escalation

The final step in artificial nutrition therapy is to consider when withholding or withdrawing. Particularly, the transition from parenteral nutrition to enteral nutrition and therefore to oral nutrition could be a slow process focused on the tolerance of different nutritional approaches up to oral feeds. In the case of enteral feeding, some patients will want to continue feeding with an intermittent or cyclic tube as they switch to oral feeding, while others may be able to make a more dramatic transition. In the case of end of life care in terminally ill patients, patients, physicians, and family members need to discuss the patient's desires, carefully weigh the benefits and burdens of tube feeding, and examine their own beliefs and biases [18].

#### D for discharge

In order to improve the outcome of critical illness, a multimodal intervention for an optimal nutrition therapy should be provided during critical illness, after ICU discharge, and following hospital discharge. Upon discharge from the ICU the patient may experience further loss of muscle and energy in the absence of good nutritional guidance and physical activity. A comprehensive multidisciplinary approach should be implemented, from ICU admission to hospital stay to rehabilitation, as increased frailty throughout the entire hospital stay can substantially impair the ability to achieve successful rehabilitation upon discharge

### Measuring the performance and outcomes of an NS program

**Fig. 2:**
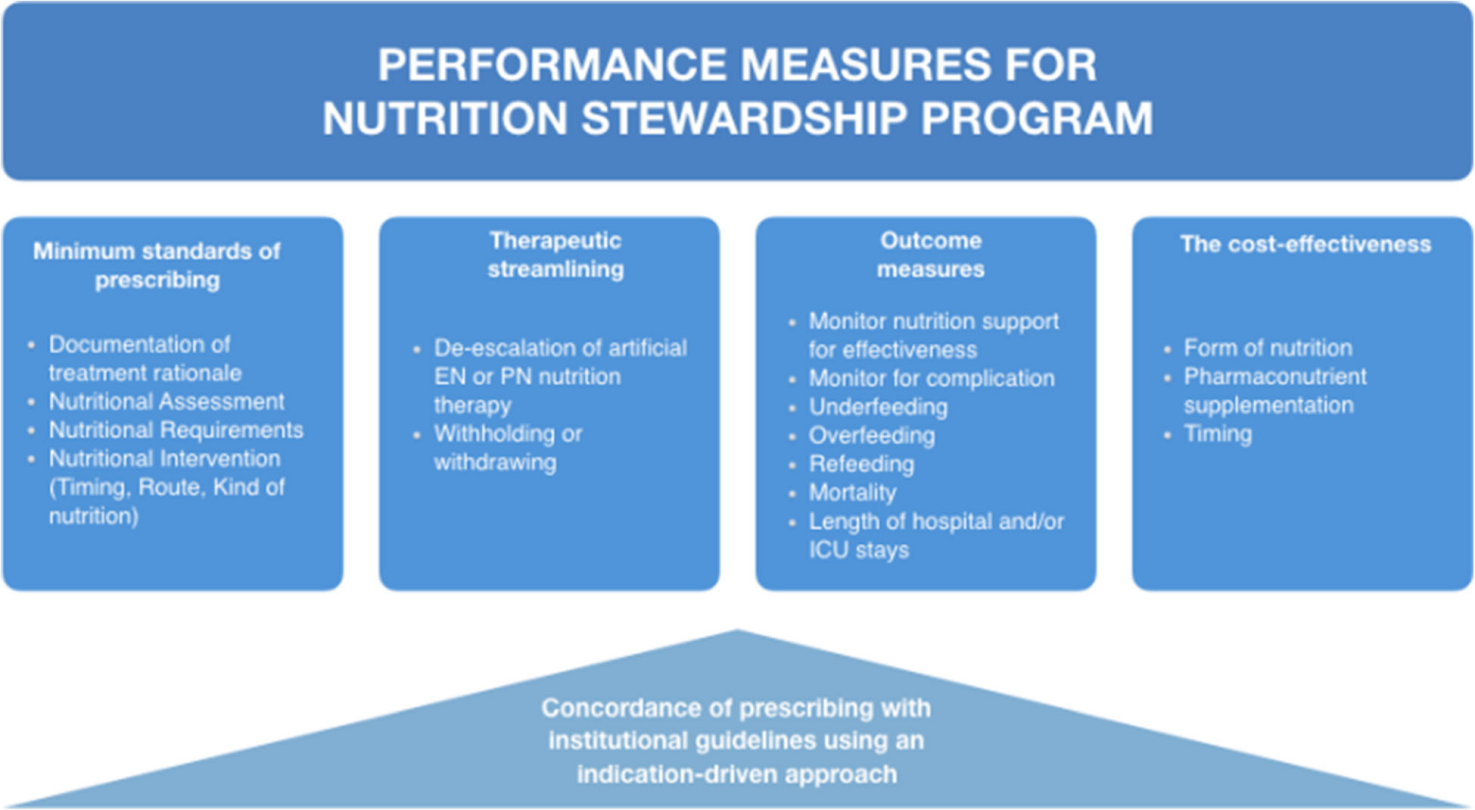
Performance measures for nutrition stewardship program. EN, enteral nutrition; ICU, intensive care unit; PN, parenteral nutrition

The ongoing benefit of an NS program must be demonstrated to the hospital medical management who performs cyclical monitoring of the facility's processes, outcomes, and measures (presented in Fig. 2) related to the prevention and management of malnutrition. Recently, Brunelli et al. [19] in their retrospective study stressed the need of monitoring nutrition prescribing behaviors in acute hospitals in order to better set up tailored interventions to standardize clinicians’ practices and to focus on specific training targets. Normally, initial goals should be somewhat modest to allow for initial successes. In addition to clinical audit, at the population level, surveys are a useful means of identifying areas that need attention. Measurement of nutritional consumption and the use of this data to benchmark institutions is problematic because of differences in case-mix or institutional practices. From an NSP perspective, maintaining high prescribing standards could be regarded as a surrogate for patient safety and improved clinical outcomes as it ensures that the most effective nutritional support is being given and that nutrition-related adverse events are being minimized. Clinical audit should be performed as a core activity and it should be evaluated if the impact of adequate nutritional intake tailored to the recovery stage of ICU patients can improve metabolic condition, decrease morbidity, and optimize long-term rehabilitation success.

## Conclusion

Critical ill patients require rapid, effective, and complete nutritional support management by a trained group of individuals. A 6D’s Framework of Nutritional Stewardship could be helpful to obtain a better clinical outcome, prevention of adverse events, and reduction cost. A multimodal intervention for an optimal nutrition therapy should be provided during critical illness, after ICU discharge, and following hospital discharges. The cornerstone of NSP is the implementation of institutional guidelines which should largely accord with international or national standards augmented by provider audit and feedback, the quality use of medicine indicators, and performance measures. A multidisciplinary team engaging and consensus building is vital for mission success.

## Data Availability

Not applicable

## Abbreviations

EN: Enteral nutrition
ESPEN: European Society for Clinical Nutrition and Metabolism
ICU: Intensive care unit
NSP: Nutrition stewardship program
